# Unraveling the Electronic Structures of Neodymium in LiLuF_4_ Nanocrystals for Ratiometric Temperature Sensing

**DOI:** 10.1002/advs.201802282

**Published:** 2019-03-14

**Authors:** Ping Huang, Wei Zheng, Datao Tu, Xiaoying Shang, Meiran Zhang, Renfu Li, Jin Xu, Yan Liu, Xueyuan Chen

**Affiliations:** ^1^ CAS Key Laboratory of Design and Assembly of Functional Nanostructures and Fujian Key Laboratory of Nanomaterials Fujian Institute of Research on the Structure of Matter Chinese Academy of Sciences Fuzhou Fujian 350002 China

**Keywords:** energy level, LiLuF_4_ nanocrystals, neodymium, site symmetry, temperature sensing

## Abstract

Nd^3+^‐doped near‐infrared (NIR) luminescent nanocrystals (NCs) have shown great promise in various bioapplications. A fundamental understanding of the electronic structures of Nd^3+^ in NCs is of vital importance for discovering novel Nd^3+^‐activated luminescent nanoprobes and exploring their new applications. Herein, the electronic structures of Nd^3+^ in LiLuF_4_ NCs are unraveled by means of low‐temperature and high‐resolution optical spectroscopy. The photoactive site symmetry of Nd^3+^ in LiLuF_4_ NCs and its crystal‐field (CF) transition lines in the NIR region of interest are identified. By taking advantage of the well‐resolved and sharp CF transition lines of Nd^3+^, the application of LiLuF_4_:Nd^3+^ NCs as sensitive NIR‐to‐NIR luminescent nanoprobes for ratiometric detection of cryogenic temperature with a linear range of 77–275 K is demonstrated. These findings reveal the great potential of LiLuF_4_:Nd^3+^ NCs in temperature sensing and also lay a foundation for future design of efficient Nd^3+^‐based luminescent nanoprobes.

## 1 Introduction

Trivalent neodymium (Nd^3+^) ion doped luminescent nanocrystals (NCs) have recently attracted considerable attention owing to their superior optical properties in the near‐infrared (NIR) spectral region.^1^ These Nd^3+^‐doped NCs are able to emit NIR light under excitation with a low‐cost 808 nm diode laser, which feature a series of advantages such as large penetration depth, minimal background interference, and little damage to the targeted samples, and thus are regarded as excellent NIR‐to‐NIR luminescent nanoprobes for various bioapplications.^2^ Specifically, Nd^3+^‐doped NCs have been frequently used as sensitive nanothermometers for physiological temperature sensing in tissues based on the temperature‐dependent energy transfer with other lanthanide (Ln^3+^) activators or the eletronic transitions from the thermally coupled crystal‐field (CF) energy levels of Nd^3+^.^3^ Nonetheless, the CF transition lines of Nd^3+^‐doped NCs are usually elusive and undistinguishable at physiological temperatures because of the line broadening and multiple sites of Nd^3+^ in NCs.^4^ As a result, the assignment of CF transition lines in previously reported Nd^3+^‐based nanothermometer had to rely on the reference of CF levels of bulk analogs,[[qv: 3b,5]] which could be unreliable and lead to an artificial detection result. Therefore, it is urgent to unravel the electronic structures of Nd^3+^ in NCs and assign its CF transition lines in the NIR of interest, which is of fundamental significance for designing novel Nd^3+^‐activated luminescent nanoprobes and exploring their new applications.

Lithium lutetium tetrafluoride (LiLuF_4_), owing to its low phonon energy and high chemical stability, is an excellent host material for Nd^3+^ doping to produce efficient upconverting and downshifting luminescence.^6^ Nd^3+^‐activated LiLuF_4_ bulk crystals have been documented as efficient solid‐state laser crystals,^7^ and their nanoscale counterparts have been reported as sensitive NIR‐to‐NIR luminescent nanoprobes for subtissue bioimaging.[[qv: 6d]] Moreover, the luminescence of Nd^3+^ in LiLuF_4_ lattice is characterized by sharp emission peaks even at room temperature due to the strong CF level splitting,[[qv: 6a]] which facilitates the discrimination of the CF transition lines of Nd^3+^ in NIR and thereby enables the assignment of CF energy levels of Nd^3+^ in nanoscale LiLuF_4_.

Herein, we report for the first time the electronic structures of Nd^3+^ in LiLuF_4_ NCs. We first use Eu^3+^ ion as the structural probe to unveil the local site symmetry of Ln^3+^ dopants in LiLuF_4_ NCs by means of high‐resolution photoluminescence (PL) spectroscopy, time‐resolved PL (TRPL) spectroscopy, and site‐selective PL spectroscopy at 10 K. With definite local site symmetry, we then assign the CF transition lines of Nd^3+^ in the NIR region and the corresponding CF levels through temperature‐dependent PL spectroscopy. Furthermore, by taking advantage of the well‐resolved CF transition lines from the thermally coupled Stark sublevels of ^4^F_3/2_ of Nd^3+^, we show the application of LiLuF_4_:Nd^3+^ NCs as NIR‐to‐NIR luminescent nanoprobes for ratiometric detection of cryogenic temperature with high reliability and sensitivity, thereby revealing the great potential of LiLuF_4_:Nd^3+^ NCs for temperature sensing.

## 2 Results and Discussion

**Figure 1:**
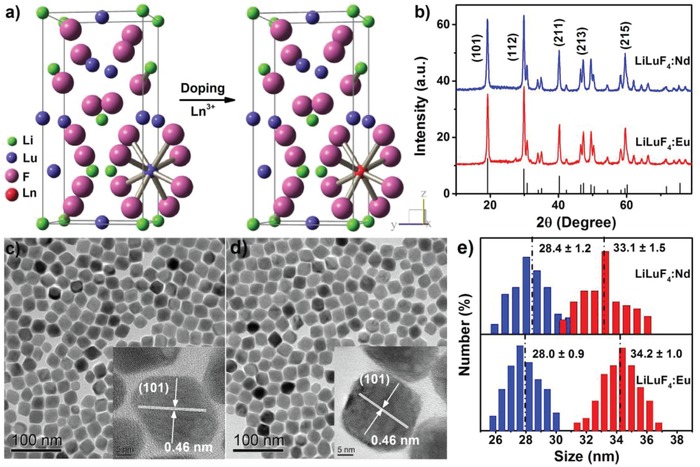
a) Crystal structure of tetragonal LiLuF_4_ and the crystallographic site for Ln^3+^ dopants. b) XRD patterns of LiLuF_4_:2%Nd^3+^ and LiLuF_4_:5%Eu^3+^ NCs. The bottom lines represent the standard XRD pattern of tetragonal LiLuF_4_ (JCPDS No. 027‐1251). c,d) TEM images of LiLuF_4_:2%Nd^3+^ and LiLuF_4_:5%Eu^3+^ NCs. The insets show the corresponding high‐resolution TEM images. e) Size distributions of the NCs obtained by randomly calculating 200 particles in the TEM images. The blue and red bars represent the width and length of the rhomboid NCs, respectively.

The LiLuF_4_ crystal has a scheelite structure (space group *I*4_1_/a) with Lu^3+^ ions surrounded by eight F^−^ ions that form the edges of a slightly distorted dodecahedron. All Lu^3+^ ions occupy a single crystallographic site of *S*_4_ symmetry (**Figure** **1**a). High‐quality LiLuF_4_:Ln^3+^ (Ln = Eu and Nd) NCs were synthesized through a thermal decomposition method as we previously reported.[[qv: 6b]] The as‐synthesized NCs are hydrophobic and can be readily dispersed in a variety of nonpolar organic solvents such as cyclohexane. X‐ray diffraction (XRD) patterns (Figure 1b) show that all diffraction peaks of the NCs can be well indexed into tetragonal LiLuF_4_ (JCPDS No. 027‐1251) without any additional impurities. Transmission electron microscopy (TEM) images show that both LiLuF_4_:Nd^3+^ and LiLuF_4_:Eu^3+^ NCs are rhomboid with mean sizes of (28.4 ± 1.2) × (33.1 ± 1.5) and (28.0 ± 0.9) × (34.2 ± 1.0) nm, respectively (Figure 1c–e). High‐resolution TEM images (insets of Figure 1c,d) exhibit clear lattice fringes with an observed d spacing of 0.46 nm for the (101) plane of tetragonal LiLuF_4_, confirming pure phase and high crystallinity of the resulting NCs. Compositional analyses through energy‐dispersive X‐ray spectrum and inductively coupled plasma‐atomic emission spectroscopy reveal 1.8 mol% of Nd^3+^ and 4.6 mol% of Eu^3+^ in LiLuF_4_ matrix (Figure S1, Supporting Information), which are generally consistent with their nominal dopant concentrations (2 mol% of Nd^3+^ and 5 mol% of Eu^3+^).

**Figure 2:**
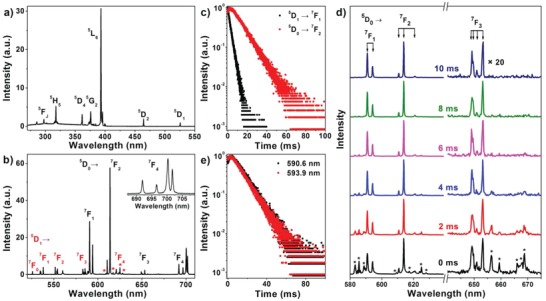
a) 10 K PL excitation spectrum of LiLuF_4_:5%Eu^3+^ NCs by monitoring the Eu^3+^ emission at 613.8 nm and b) their emission spectrum upon excitation at 393.0 nm. The inset in (b) enlarges the ^5^D_0_→^7^F_4_ emissions including four CF transition lines, and the asterisks represent the ^5^D_1_→^7^F_4_ emissions of Eu^3+^. c) PL decay curves of LiLuF_4_:5%Eu^3+^ NCs by monitoring the ^5^D_0_→^7^F_2_ and ^5^D_1_→^7^F_1_ emissions of Eu^3+^ at 613.8 and 582.8 nm, respectively. d) 10 K TRPL spectra of LiLuF_4_:5%Eu^3+^ NCs with different delay times under excitation at 393.0 nm. The asterisks denote the CF transition lines from the ^5^D_1_ multiplet of Eu^3+^. e) PL decay curves of LiLuF_4_:5%Eu^3+^ NCs by monitoring the ^5^D_0_→^7^F_1_ emissions of Eu^3+^ at 590.6 and 593.9 nm.

Eu^3+^ ion is a sensitive spectroscopic probe, which can provide site symmetry information because of its nondegenerate emissive state of ^5^D_0_ and the ground state of ^7^F_0_.^8^ To probe the practical local site symmetry of Ln^3+^ dopants in LiLuF_4_ NCs, we measured the high‐resolution PL spectra of Eu^3+^ in LiLuF_4_ NCs. Emission and excitation spectra and PL decays were recorded at 10 K to avoid thermal broadening of spectral lines at room temperature (Figure S2, Supporting Information).^9^ **Figure** **2** shows the high‐resolution PL spectra of LiLuF_4_:5%Eu^3+^ NCs at 10 K, which enables a detailed assignment of the CF transition lines of Eu^3+^. By monitoring the Eu^3+^ emission at 613.8 nm, a series of CF transition lines of Eu^3+^ from the ^7^F_0_ ground state to the excited multiplets (^5^D_J_, ^5^L_J_, ^5^G_J_, ^5^H_J_, and ^5^F_J_) were observed (Figure 2a).^10^ Upon excitation to ^5^L_6_ of Eu^3+^ at 393.0 nm, the CF emission peaks from ^5^D_0_ and ^5^D_1_ to ^7^F_J_ (*J* = 0, 1, 2, 3, and 4) with full‐width at half‐maximum (FWHM) smaller than 0.5 nm were detected (Figure 2b). PL decay measurements show that both ^5^D_0_ and ^5^D_1_ display a single exponential decay with PL lifetimes of 11.3 and 2.7 ms, respectively (Figure 2c), suggesting a homogeneous CF environment around Ln^3+^ dopants in LiLuF_4_ lattice.^11^ The distinct PL lifetimes of ^5^D_0_ and ^5^D_1_ allow us to distinguish the emission peaks of ^5^D_0_ from those of ^5^D_1_ by means of TRPL spectroscopy. Figure 2d shows the TRPL spectra of LiLuF_4_:5%Eu^3+^ NCs at 10 K with different delay times. It was observed that the emission peaks from the short‐lived ^5^D_1_ level declined gradually with increasing the delay time and totally vanished when the delay time was longer than 6 ms, while the emission peaks from the long‐lived ^5^D_0_ level remained explicitly observed in the TRPL spectra even at a delay time of 10 ms. As a result, total numbers of 0, 2, 3, 4, and 4 CF transition lines of Eu^3+^ from ^5^D_0_ to ^7^F_0_, ^7^F_1_, ^7^F_2_, ^7^F_3_, and ^7^F_4_ can be discerned in LiLuF_4_ NCs. To check whether all these transition lines arise from the same site, site‐selective excitation spectra were measured by monitoring the three peaks of ^5^D_0_→^7^F_2_ at 610.4, 613.8, and 620.8 nm. The obtained excitation spectra were coincident (Figure S3, Supporting Information), indicating that the PL originated from Eu^3+^ ions occupying a single spectroscopic site, as also evidenced by the essentially identical site‐selective emission spectra upon excitation to ^5^L_6_ at 393.0, 395.8, 399.4, and 400.6 nm and the same PL lifetimes of the ^5^D_0_→^7^F_1_ emissions at 590.6 and 593.9 nm (Figure 2e and Figure S4, Supporting Information). According to the branching rules and the transition selection rules of the 32 point groups (Table S1, Supporting Information),[[qv: 8b,c]] the spectroscopic site symmetry of Eu^3+^ in LiLuF_4_ NCs was determined to be *S*_4_, which agrees well with the crystallographic site symmetry of Lu^3+^ in LiLuF_4_. These results suggest that Ln^3+^ ions are prone to occupy a single spectroscopic site of *S*_4_ symmetry in LiLuF_4_ NCs at low doping levels (<5 mol%) due to the close ionic radii and chemical properties of Ln^3+^ ions.

**Figure 3:**
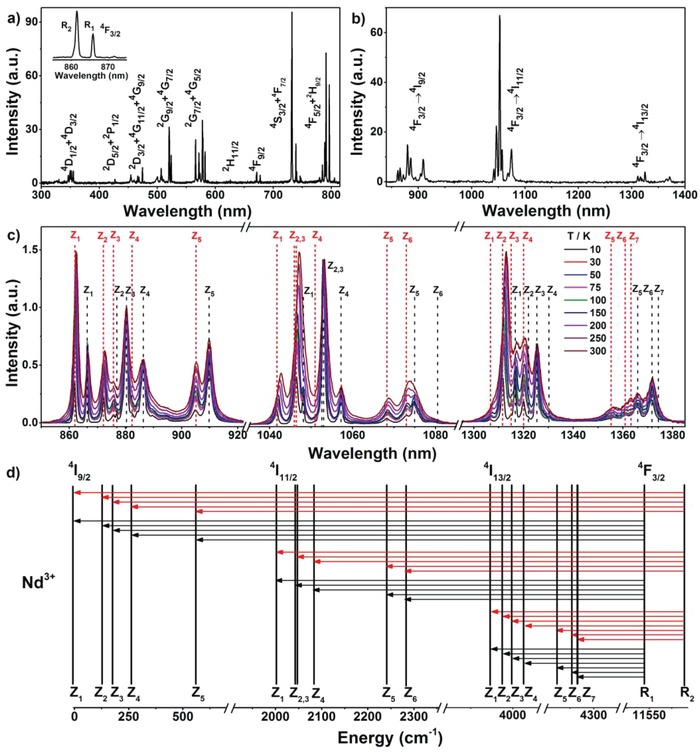
a) 10 K PL excitation spectrum of LiLuF_4_:2%Nd^3+^ NCs by monitoring the Nd^3+^ emission at 1053.2 nm and b) their emission spectrum upon excitation at 791.3 nm. The inset in (a) shows two CF transition lines from the ^4^I_9/2_ ground state to the upper and lower Stark sublevels of ^4^F_3/2_. c) Temperature‐dependent PL emission spectra (10–300 K) for the ^4^F_3/2_→^4^I_J_ (*J* = 9/2, 11/2, and 13/2) CF transitions of Nd^3+^ in LiLuF_4_ NCs upon 808 nm diode laser excitation at a power density of 1 W cm^−2^. The spectra were normalized at the maximum intensities around 880.4, 1053.1, and 1325.1 nm for the emissions from ^4^F_3/2_ to ^4^I_9/2_, ^4^I_11/2_, and ^4^I_13/2_, respectively. The dashed lines denote the CF transitions from the R_1_ (black) and R_2_ (red) Stark sublevels of ^4^F_3/2_ to those of ^4^I_J_. d) CF energy levels of the ^4^F_3/2_ and ^4^I_J_ multiplets of Nd^3+^ in LiLuF_4_ NCs, showing all CF transitions observed in (c).

With definite local site symmetry of Ln^3+^ dopants, we are able to assign the CF transition lines of Nd^3+^ in LiLuF_4_ NCs by means of high‐resolution PL spectroscopy. **Figure** **3**a shows the PL excitation spectrum of Nd^3+^ in LiLuF_4_ NCs at 10 K by monitoring the Nd^3+^ emission at 1053.2 nm, from which a series of CF transition lines of Nd^3+^ from the ^4^I_9/2_ ground state to the excited multiplets (^4^F_J_, ^2^H_J_, ^4^S_J_, ^2^G_J_, ^4^G_J_, ^2^D_J_, and ^4^D_J_) were identified.^12^ Specifically, two excitation peaks at 861.4 and 865.9 nm were clearly observed (inset of Figure 3a), ascribing to the CF transitions of Nd^3+^ from the ^4^I_9/2_ ground state to the upper (R_2_) and lower (R_1_) Stark sublevels of ^4^F_3/2_, respectively. This implies that the CF levels of Nd^3+^ are doubly degenerate in LiLuF_4_ NCs, as expected for a Kramers ion.^13^ 10 K PL emission spectrum (Figure 3b) shows that the NCs exhibit a set of characteristic and sharp emission peaks (FWHM < 0.9 nm) from the two Stark sublevels of ^4^F_3/2_ (R_1_ and R_2_) to those of ^4^I_9/2_, ^4^I_11/2_, and ^4^I_13/2_ of Nd^3+^ under xenon lamp excitation at 791.3 nm. To confirm that all these transition lines arise from a single site, we recorded PL emission spectrum of the NCs by exciting them with an 808 nm diode laser, whereby all possible spectroscopic sites of Nd^3+^ could be excited in view of the high power density (50 W cm^−2^) and relatively wide FWHM (3.2 nm) of the laser source (Figure S5, Supporting Information). It turned out that the emission pattern of the NCs under 808 nm diode laser excitation was exactly identical to that under xenon lamp excitation at 791.3 nm (Figure S6, Supporting Information), inferring that the PL originates from Nd^3+^ ions occupying a single spectroscopic site. From the emission spectrum, total numbers of 10, 12, and 14 CF transition lines of Nd^3+^ from ^4^F_3/2_ to ^4^I_9/2_, ^4^I_11/2_, and ^4^I_13/2_ were discerned, which agree well with the theoretically predicted numbers for Nd^3+^ in LiLuF_4_ with doubly degenerate CF levels.^13^ These results demonstrate unambiguously that Nd^3+^ ions occupy a single spectroscopic site of *S*_4_ symmetry in LiLuF_4_ NCs, as revealed by using Eu^3+^ as the structural probe.

Because the energy difference between the R_1_ and R_2_ Stark sublevels of ^4^F_3/2_ is only 58 cm^−1^, the higher Stark sublevel (R_2_) is easily thermally populated from the lower one (R_1_). As a result, PL intensity ratio between the emissions from R_2_ and R_1_ would increase with the temperature rise, enabling discrimination of the CF transition lines of R_2_ from those of R_1_ through the temperature‐dependent PL emission spectra (Figure 3c). It was observed that the intensities of the CF emission peaks at 861.9, 872.2, 875.7, 882.3, and 904.9 nm increased significantly with the temperature rise, corresponding to the transitions from the R_2_ sublevel of ^4^F_3/2_ to the five CF levels (Z_1_, Z_2_, Z_3_, Z_4_, and Z_5_) of ^4^I_9/2_ (Figure 3d).[[qv: 12c]] By contrast, the intensities of the CF emission peaks at 866.2, 876.9, 880.4, 886.4, and 909.8 nm showed only a slight increase with the temperature rise, corresponding to the transitions from the R_1_ sublevel of ^4^F_3/2_. The slight increase in R_1_ lines is caused by the spectral overlap with R_2_ lines. Based on the defined CF transition lines, the CF levels of ^4^I_9/2_ can be unequivocally identified, as listed in **Table** **1**. Similarly, CF transition lines from the R_1_ and R_2_ sublevels of ^4^F_3/2_ to those of ^4^I_11/2_ and ^4^I_13/2_ can be specified by virtue of the temperature‐dependent PL emission spectra, from which the CF levels of ^4^I_11/2_ and ^4^I_13/2_ were experimentally assigned (Table 1). Besides, we also found a redshift in CF transition lines of Nd^3+^ with the temperature rise, especially for the transitions from R_2_ of ^4^F_3/2_ to Z_1_ and Z_2_ of ^4^I_11/2_ (Figure 3c), as a result of enhanced electron‐phonon coupling at higher temperatures.[[qv: 4b]] Importantly, we found that the CF transition lines from the thermally coupled R_1_ and R_2_ Stark sublevels of ^4^F_3/2_ to Z_1_ of ^4^I_9/2_ at 862 nm (R_2_→Z_1_) and 866 nm (R_1_→Z_1_) are well resolved with little interference from other Stark components at temperatures below 300 K. This feature makes LiLuF_4_:Nd^3+^ NCs an ideal nanoprobe candidate for ratiometric luminescent detection of temperature below 300 K by using the temperature‐dependent PL intensity ratio between the R_2_→Z_1_ and R_1_→Z_1_ transitions at 862 and 866 nm (*I*_862_/*I*_866_), respectively.

**Table 1:** Experimental energy levels for the ^4^I_J_ and ^4^F_3/2_ multiplets of Nd^3+^ in LiLuF_4_ NCs

Multiplet	Energy [cm^−1^]	Multiplet	Energy [cm^−1^]	Multiplet	Energy [cm^−1^]	Multiplet	Energy [cm^−1^]
^4^I_9/2_	0	^4^I_11/2_	2003	^4^I_13/2_	3948	^4^F_3/2_	11 544
	138		2041		3978		11 602
	183		2047		3996		
	265		2086		4027		
	549		2241		4222		
			2283		4254		
					4266		

**Figure 4:**
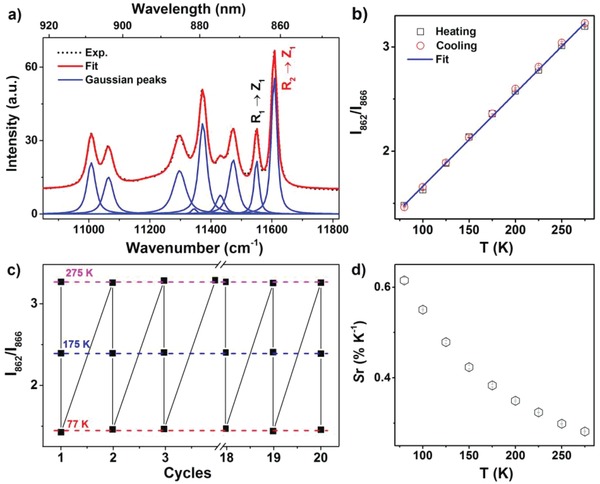
a) PL emission spectrum for the ^4^F_3/2_→^4^I_9/2_ transitions of Nd^3+^ in LiLuF_4_ NCs at 275 K and its Gaussian fit according to the CF transitions. b) PL intensity ratio between the R_2_→Z_1_ and R_1_→Z_1_ CF transitions at 862 and 866 nm (*I*_862_/*I*_866_) as a function of temperature during a heating and cooling cycle between 77 and 275 K. Each data point represents the mean (±standard deviation) of three independent measurements. c) Variation of the intensity ratio *I*_862_/*I*_866_ recorded at 77, 175, and 275 K measured over a span of 20 cycles of heating and cooling processes. d) The relative temperature sensitivity (*S*_r_) of LiLuF_4_:2%Nd^3+^ nanoprobes as a function of temperature. The error bars result from error propagation in the determination of *S*_r_.

To validate the applicability of LiLuF_4_:Nd^3+^ NCs for temperature sensing, we deconvoluted the ^4^F_3/2_→^4^I_9/2_ emission spectra of Nd^3+^ into ten Gaussian components according to the CF transitions between their Stark sublevels (**Figure** **4**a), from which the PL intensity ratio between R_2_→Z_1_ and R_1_→Z_1_ (*I*_862_/*I*_866_) was derived. Further temperature‐correlated PL emission spectra showed that the PL intensity ratio *I*_862_/*I*_866_ increased gradually with increasing the temperature from 77 to 575 K (Figure S7, Supporting Information), as a result of enhanced thermal population of the R_2_ sublevel from the R_1_ sublevel of ^4^F_3/2_ at higher temperatures. Specifically, the ratio of *I*_862_/*I*_866_ displayed a linear dependence on temperature in the range of 77–275 K, with its value increased from 1.46 at 77 K to 3.23 at 275 K (Figure 4b). Moreover, such temperature evolution of *I*_862_/*I*_866_ was found to be reversible during the heating and cooling cycle between 77 and 275 K. The ratios of *I*_862_/*I*_866_ recorded at 77, 175, and 275 K were nearly unchanged with deviations smaller than 0.5% over a span of 20 cycles of heating and cooling processes (Figure 4c), as a merit of high photochemical stability of the NCs.^14^ This indicates that the PL of LiLuF_4_:Nd^3+^ NCs is fully reversible without any observable thermal hysteresis in the temperature range of 77–275 K (Figure S8, Supporting Information), which is of key importance for temperature sensing by using a luminescent nanoprobe.^15^ The absolute temperature sensitivity (*S*_a_) of the nanoprobe, defined as the change of response *R* with temperature, namely, ∂*R*/∂T where *R* is the PL intensity ratio I_862_/I_866_ and T the absolute temperature,^16^ was calculated to be a constant of 0.00913 K^−1^, which is among the highest *S*_a_ values for Nd^3+^‐activated luminescent nanothermometer ever reported.[[qv: 3b,17]] The relative temperature sensitivity (*S*_r_), defined as the fractional rate of the change of response *R* with temperature, namely, (1/*R*)(∂*R*/∂*T*),^16^ was plotted in Figure 4d, from which the highest *S*_r_ was determined to be 0.62% K^−1^ at 77 K. The highest *S*_r_ obtained in LiLuF_4_:Nd^3+^ NCs is comparable to the best *S*_r_ values for Nd^3+^‐activated luminescent nanothermometers previously reported (Table S2, Supporting Information).^18^ The temperature uncertainty (*δT*), defined as the relative error of the response (*δR*/*R*) versus the relative temperature sensitivity (*S*_r_),[[qv: 1i,19]] was calculated to be lower than 0.6 K for temperatures below 250 K (Figure S9, Supporting Information). It is worth mentioning that the spectral overlap between different Stark or CF components in the emission spectrum of Nd^3+^ is unavoidable at high temperatures because of the CF line broadening and shifting and the multiple sites of Nd^3+^ with distinct CF surroundings in NCs. Therefore, the assignment of CF transition lines in Nd^3+^‐doped NCs should be judiciously carried out by taking into account the interference from different CF or Stark components, which is a prerequisite for temperature sensing by using Nd^3+^‐doped luminescent nanoprobes. In this regard, the superior features combined with the single photoactive site symmetry, well‐resolved CF transition lines, and high photostability of LiLuF_4_:Nd^3+^ NCs, make them excellent NIR‐to‐NIR luminescent nanoprobes for ratiometric temperature sensing in practical application.

## 3 Conclusions

In summary, we have systematically investigated the local site symmetry and electronic structures of Nd^3+^ in LiLuF_4_ NCs through low‐temperature and high‐resolution PL spectroscopy. By employing Eu^3+^ as the structural probe, a single spectroscopic site of *S*_4_ symmetry for Ln^3+^ dopants was identified in LiLuF_4_ NCs, which is consistent with the crystallographic site symmetry of Lu^3+^ in LiLuF_4_ lattice. By means of temperature‐dependent PL spectroscopy, a total number of 36 CF transition lines of Nd^3+^ in LiLuF_4_ NCs in the NIR region were unequivocally assigned. Furthermore, by employing the sharp and well‐resolved CF transitions from the thermally coupled Stark sublevels of ^4^F_3/2_ of Nd^3+^, we have demonstrated the application of LiLuF_4_:Nd^3+^ NCs as sensitive NIR‐to‐NIR luminescent nanoprobes for ratiometric detection of temperature with a wide linear range of 77–275 K. The unambiguous revelation of photoactive site symmetry and electronic structures of Nd^3+^ in inorganic NCs is of vital importance for future design and development of Nd^3+^‐based NIR luminescent nanoprobes toward versatile applications such as cryogenic temperature sensing for space and energy exploration.

## 4 Experimental Section

*Chemicals and Materials*: Lu_2_O_3_ (99.99%), Eu_2_O_3_ (99.99%), Nd_2_O_3_ (99.99%), oleic acid (OA, 90%), oleylamine (OAm, 90%), and 1‐octadecence (ODE, 90%) were purchased from Sigma‐Aldrich (Shanghai, China). CF_3_COOLi·H_2_O (99.99%), trifluoroacetic acid (≥99.0%), ethanol (≥99.5%), acetone (≥ 99.5%), and cyclohexane (≥99.5%) were purchased from Sinopharm Chemical Reagent Co. (Shanghai, China). Lu(CF_3_COO)_3_, Eu(CF_3_COO)_3_, and Nd(CF_3_COO)_3_ were prepared by dissolving Lu_2_O_3_, Eu_2_O_3_, and Nd_2_O_3_, respectively, in trifluoroacetic acid at 90 °C. All the chemical reagents were used as received without further purification.

*Synthesis of LiLuF_4_:Ln^3+^ NCs*: Monodisperse LiLuF_4_:Ln^3+^ NCs (Ln = Eu or Nd) were synthesized through a thermal decomposition method. In a typical synthesis of LiLuF_4_:2%Nd^3+^ NCs, 1 mmol of CF_3_COOLi·H_2_O, 0.98 mmol of Lu(CF_3_COO)_3_, and 0.02 mmol of Nd(CF_3_COO)_3_ were mixed with 6 mL of OA, 2 mL of OAm, and 2 mL of ODE in a 100 mL three‐neck round‐bottom flask. The resulting mixture was heated to 120 °C under N_2_ flow with constant stirring for 30 min to form a clear yellowish solution. Thereafter, the resulting solution was heated to 320 °C under N_2_ flow with vigorous stirring for 40 min and then cooled down to room temperature. The obtained NCs were precipitated by addition of 20 mL of acetone, collected by centrifugation, washed with ethanol several times, and finally dried in vacuum at 60 °C for 24 h.

*Structural and Optical Characterization*: Powder XRD patterns of the samples were collected with an X‐ray diffractometer (MiniFlex2, Rigaku) using Cu Kα1 radiation (λ = 0.154187 nm). Both the low‐ and high‐resolution TEM measurements were performed by using a TECNAI G^2^ F20 TEM equipped with an energy‐dispersive X‐ray spectrum. Inductively coupled plasma (ICP) analysis was conducted by using Inductively Coupled Plasma AES spectrometer (Ultima2, Jobin Yvon). PL excitation and emission spectra and PL decays were recorded on an Edinburgh Instruments FLS920 spectrometer equipped with both continuous (450 W) and pulsed xenon lamp at room temperature. For low temperature measurement, samples were mounted on a closed cycle cryostat (10–350 K, DE202, Advanced Research Systems). The emission or excitation monochromator's slits were set as small as possible to maximize the instrumental resolution, and the highest wavelength resolution is 0.05 nm. The line intensities and positions of the measured spectra were calibrated according to the correction curve of the instrument and standard mercury lamp.

## Conflict of Interest

The authors declare no conflict of interest.

## Supporting information

Supplementary
