# Supplementary material for: Unraveling the Electronic Structures of Neodymium in LiLuF4 Nanocrystals for Ratiometric Temperature Sensing
Source: Adv Sci (Weinh). 2019 Mar 14;6(10):1802282. doi: 10.1002/advs.201802282 (PMC6523367; doi:10.1002/advs.201802282)
Supplement: Supplementary file 1 — Supplementary [file ADVS-6-1802282-s001.pdf]

Copyright WILEY-VCH Verlag GmbH & Co. KGaA, 69469 Weinheim, Germany, 2019.

ADVANCED SCIENCE
Open Access

Supporting Information

for *Adv. Sci.,* DOI: 10.1002/advs.201802282

Unraveling the Electronic Structures of Neodymium in $LiLuF_4$ Nanocrystals for Ratiometric Temperature Sensing

Ping Huang, Wei Zheng, Datao Tu, Xiaoying Shang, Meiran Zhang, Renfu Li, Jin Xu, Yan Liu, and Xueyuan Chen**

Copyright WILEY-VCH Verlag GmbH & Co. KGaA, 69469 Weinheim, Germany, 2019.

Supporting Information

Unraveling the electronic structures of neodymium in $LiLuF_4$ nanocrystals for ratiometric temperature sensing

Ping Huang, Wei Zheng*, Datao Tu, Xiaoying Shang, Meiran Zhang, Renfu Li, Jin Xu, Yan Liu, and Xueyuan Chen*

CAS Key Laboratory of Design and Assembly of Functional Nanostructures, and Fujian Key Laboratory of Nanomaterials, Fujian Institute of Research on the Structure of Matter, Chinese Academy of Sciences, Fuzhou, Fujian 350002, China.

Fax: +86-591-63179421; Tel: +86-591-63179421;

E-mail: zhengwei@fjirsm.ac.cn or xchen@fjirsm.ac.cn

Table S1. Theoretically allowed transition lines of $^5D_0 \rightarrow ^7F_J$ of Eu^{3+} at 32 crystallographic point groups.

Local symmetry	Point group	The number of allowed transition lines of $^5D_0 \rightarrow ^7F_J$						
		**0**	**1**	**2**	**3**	**4**	**5**	**6**
**Triclinic**	C_1	1	3	5	7	9	11	13
	C_i	0	3	0	0	0	0	0
**Monoclinic**	C_s	1	3	5	7	9	11	13
	C_2	1	3	5	7	9	11	13
	C_{2h}	0	3	0	0	0	0	0
**Orthorhomic**	C_{2v}	1	3	4	5	7	8	10
	D_2	0	3	3	6	6	9	9
	D_{2h}	0	3	0	0	0	0	0
**Tetragonal**	C_4	1	2	2	3	5	6	6
	C_{4v}	1	2	2	2	4	4	5
	S_4	0	2	3	4	4	5	7
	D_{2d}	0	2	2	3	3	4	5
	D_4	0	2	1	3	3	5	4
	C_{4h}	0	2	0	0	0	0	0
	D_{4h}	0	2	0	0	0	0	0
**Trigonal**	C_3	1	2	3	5	6	7	9
	C_{3v}	1	2	3	3	5	5	7
	D_3	0	2	2	4	4	6	6
	D_{3d}	0	2	0	0	0	0	0

	**S_6**	0	2	0	0	0	0	0
**Hexagonal**	**C_6**	1	2	2	2	2	3	5
	**C_{6v}**	1	2	2	1	2	2	4
	**D_6**	0	2	1	2	1	3	3
	**C_{3h}**	0	2	1	3	4	4	4
	**D_{3h}**	0	2	1	2	3	3	3
	**C_{6h}**	0	2	0	0	0	0	0
	**D_{6h}**	0	2	0	0	0	0	0
**Cubic**	**T**	0	1	1	2	2	3	3
	**T_d**	0	1	1	1	1	1	2
	**T_h**	0	1	0	0	0	0	0
	**O**	0	1	0	1	1	2	1
	**O_h**	0	1	0	0	0	0	0

Table S2. Representative temperature sensing based on Nd^{3+}-activated luminescent nanothermometers: materials, fluorescence intensity ratio (FIR) as the detection signal, detection temperature range, and relative temperature sensitivity (S_r).

Materials	FIR	Temperature range (K)	S_r (% K^{-1})	Ref.
$LiLuF_4$:Nd	I_{862}/I_{866}	77-275	0.62	This work
Gd_2O_3:Nd	$I_{782-865}/I_{865-925}$	288-323	1.75	[1]
SrF_2:Gd,Nd	I_{859}/I_{867}	293-338	0.61	[2]
SrF_2:Gd,Nd	$I_{1000-1150}/I_{867}$	293-338	0.50	[2]
YVO_4:Nd@SiO_2	$I_{1064.7}/I_{1066.3}$	298-333	0.40	[3]
LaF_3:Nd	I_{885}/I_{863}	303-333	0.26	[4]
$LiNdP_4O_{12}$	I_{870}/I_{866}	250–500	0.25	[5]
$LiYF_4$:Nd @$LiYF_4$@$LiYF_4$:Nd	I_{1046}/I_{1056}	293-323	0.22	[6]
CaF_2:Y,Nd	I_{1053}/I_{1062}	296-333	0.18	[7]
$KGd(WO_4)_2$:Nd	I_{1076}/I_{1068}	293-333	0.16	[8]
$KGd(WO_4)_2$:Nd	I_{896}/I_{884}	293-333	0.12	[8]
$NaYF_4$:Nd	I_{863}/I_{870}	273-423	0.12	[9]

a)
b)

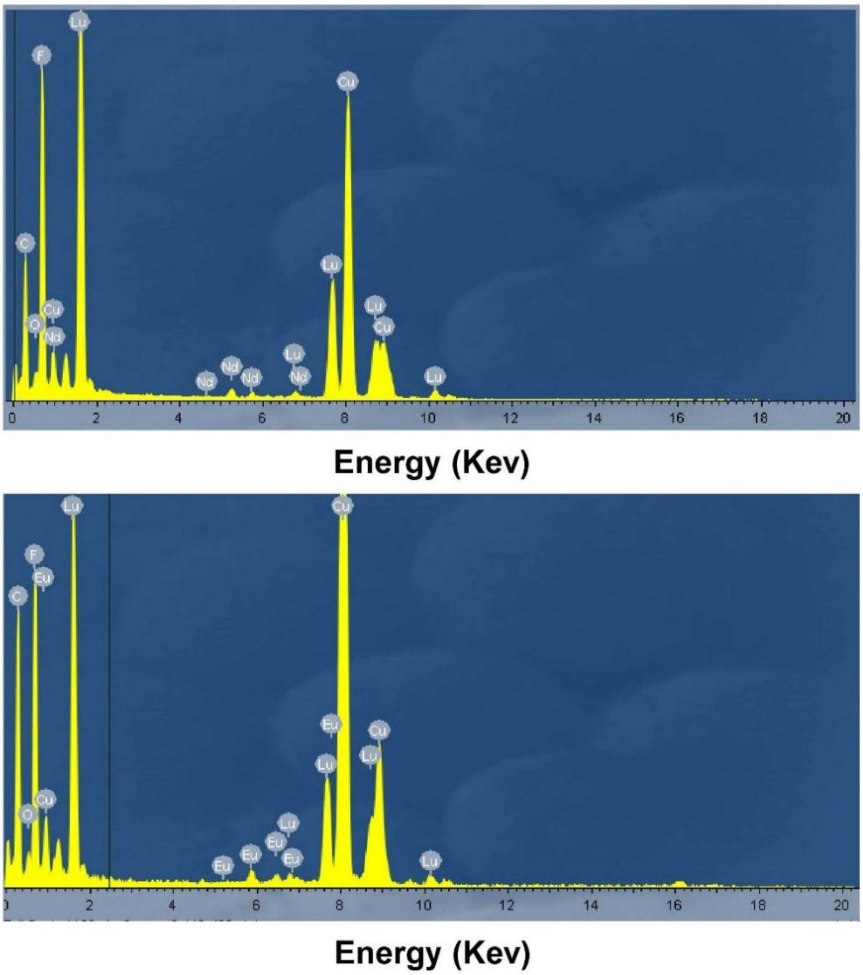

Figure S1. Energy-dispersive X-ray spectra of a) $LiLuF_4$:2%Nd^{3+} and b) $LiLuF_4$:5%Eu^{3+} NCs, showing the elements of Lu, F and Nd in $LiLuF_4$:2%Nd^{3+} NCs, and Lu, F and Eu in $LiLuF_4$:5%Eu^{3+} NCs. The absence of Li is because of its small atomic number that cannot be detected by energy-dispersive X-ray spectra.

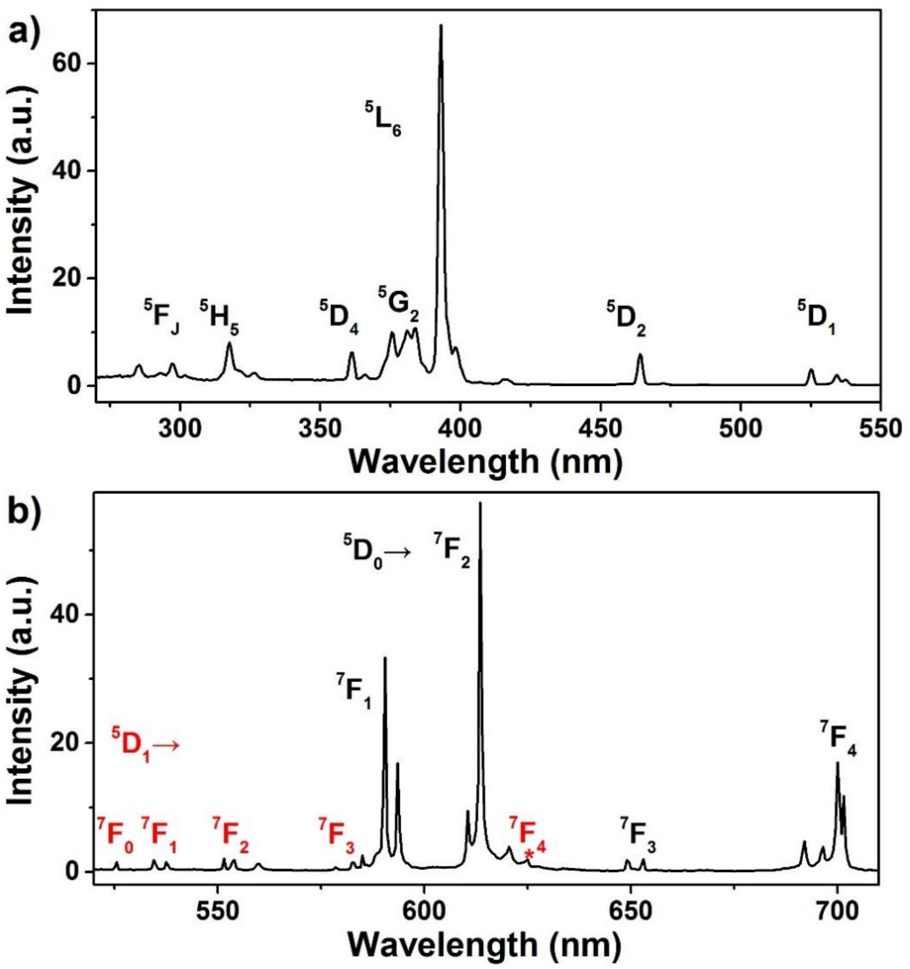

Figure S2. Room temperature a) PL excitation (λ_{em} = 614 nm) and b) emission (λ_{ex} = 393 nm) spectra of $LiLuF_4$:5%Eu^{3+} NCs. The spectral lines of Eu^{3+} are broadened at room temperature in comparison with those at 10 K.

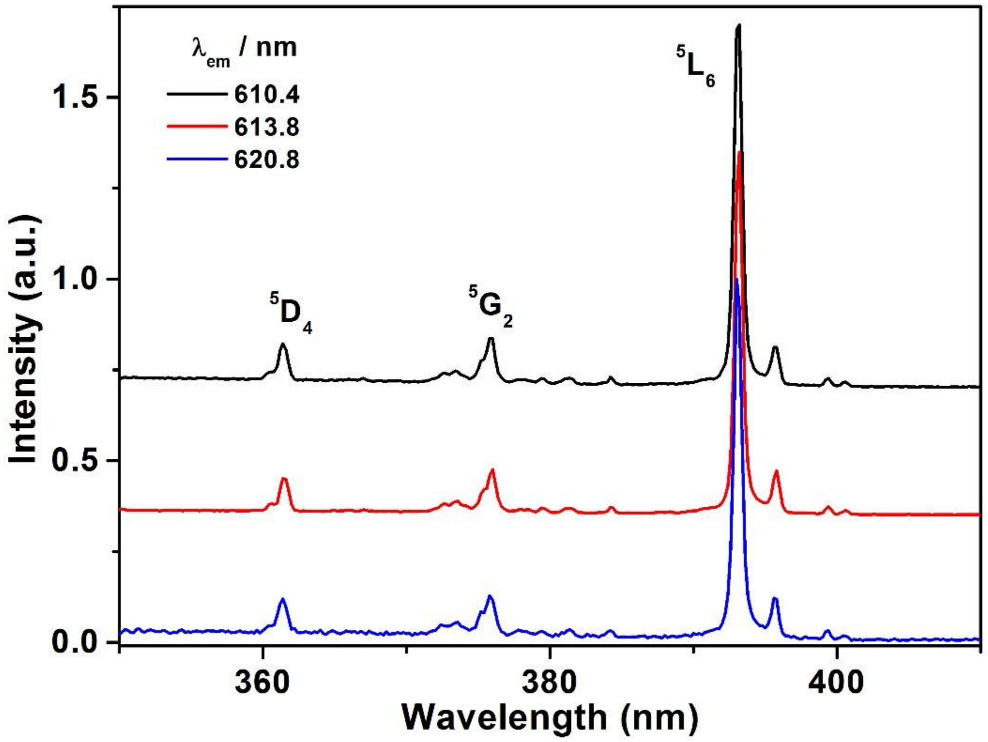

Figure S3. Site-selective PL excitation spectra of $LiLuF_4$:5%Eu^{3+} NCs by monitoring the three peaks of $^5D_0 \rightarrow {}^7F_2$ transitions of Eu^{3+} at 610.4, 613.8 and 620.8 nm at 10 K. All the spectra are coincident, suggesting that the PL originated from Eu^{3+} ions occupying the same kind of spectroscopic site.

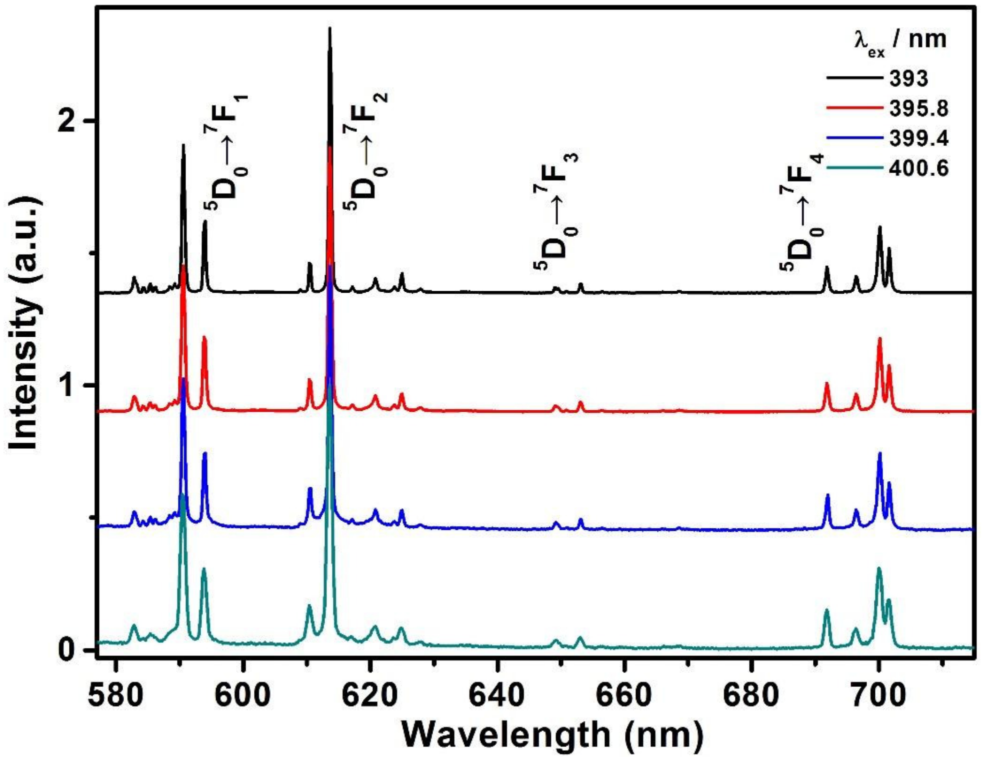

Figure S4. Site-selective PL emission spectra of $LiLuF_4$:5%Eu^{3+} NCs upon excitation to $^5L_6$ of Eu^{3+} at 393.0, 395.8, 399.4 and 400.6 nm at 10 K. All the spectra are coincident, confirming that the PL originated from Eu^{3+} ions occupying a single spectroscopic site.

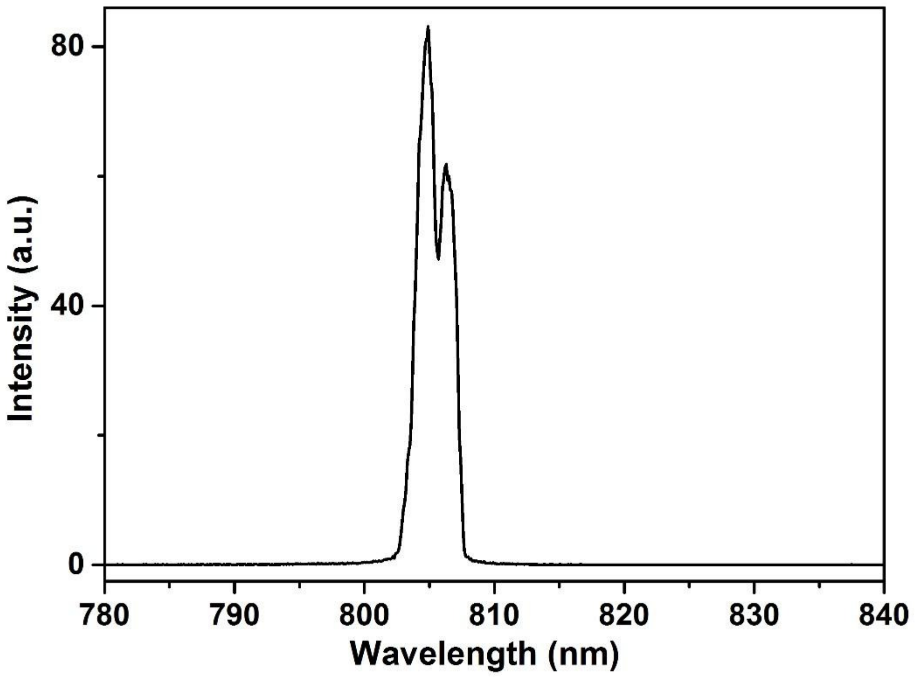

Figure S5. Emission spectrum of the 808-nm diode laser measured at a power density of 50 W cm^{-2} by using an attenuator. The peak wavelength of the laser locates at 805 nm, and the full-width at half-maximum of the laser is approximately 3.2 nm.

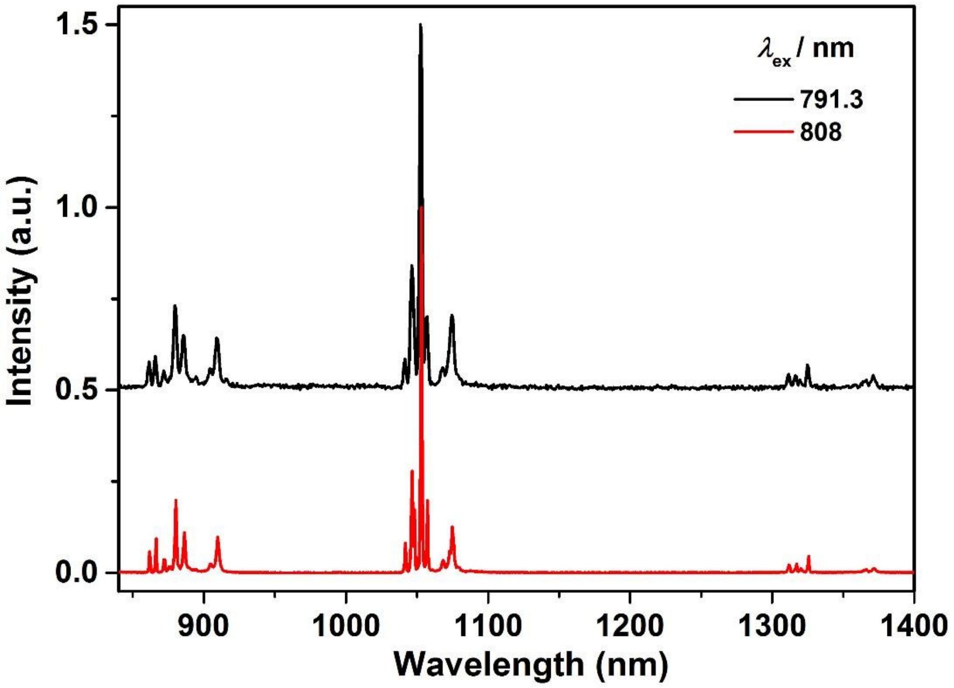

Figure S6. 10 K PL emission spectra of $LiLuF_4$:2%Nd^{3+} NCs under excitation with a xenon lamp at 791.3 nm and an 808-nm diode laser (50 W cm^{-2}), respectively. The emission pattern of the NCs under 808-nm diode laser excitation is exactly identical to that under xenon lamp excitation at 791.3 nm, inferring that the PL originates from Nd^{3+} ions occupying a single spectroscopic site.

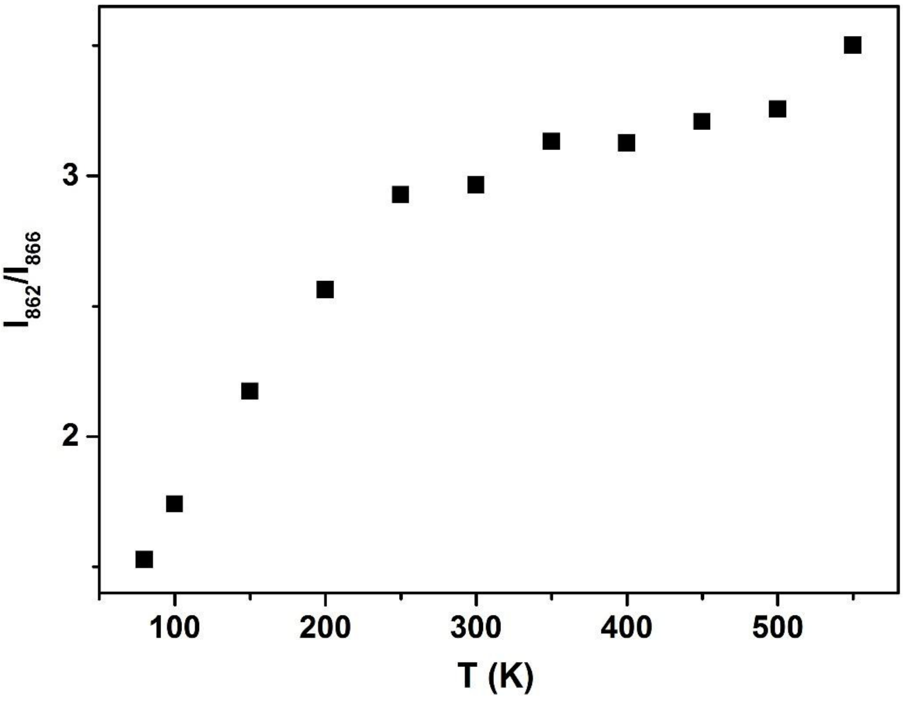

Figure S7. PL intensity ratio between the $R_2 \rightarrow Z_1$ and $R_1 \rightarrow Z_1$ CF transitions for $^4F_{3/2} \rightarrow ^4I_{9/2}$ of Nd^{3+} at 862 and 866 nm (I_{862}/I_{866}) in $LiLuF_4$:2%Nd^{3+} NCs as a function of temperature, showing a gradual increase I_{862}/I_{866} value with the temperature rise, as a result of enhanced thermal population of the R_2 sublevel from the R_1 sublevel of $^4F_{3/2}$ of Nd^{3+} at higher temperatures.

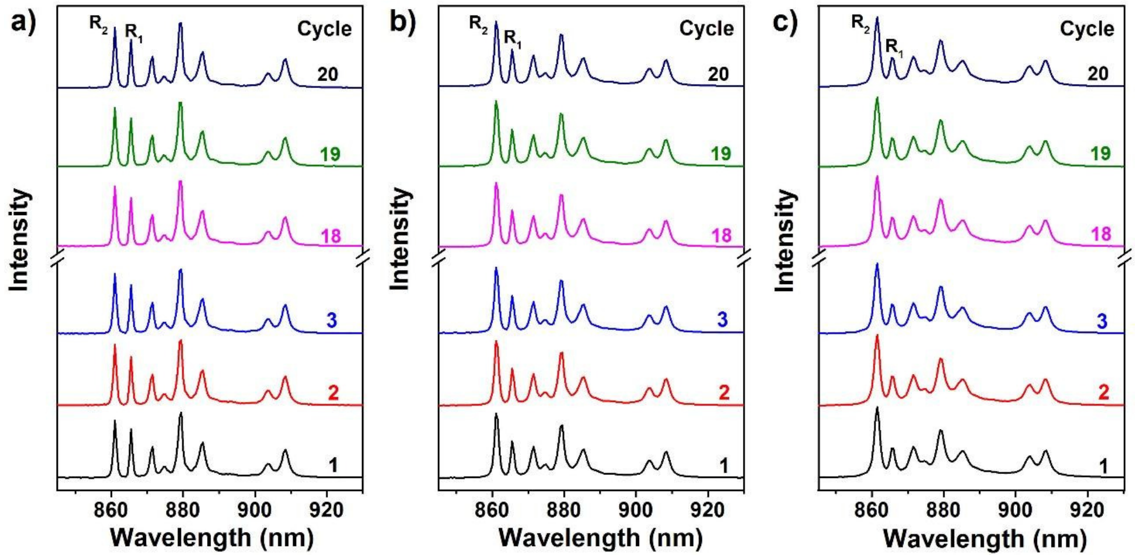

Figure S8. PL emission spectra of $LiLuF_4$:2%Nd^{3+} NCs recorded at a) 77, b) 175, and c) 275 K under 808-nm diode laser excitation with a power density of 1 W cm^{-2} over a span of 20 cycles of heating and cooling processes. The spectra are nearly unchanged during the heating and cooling processes, indicative of a high photochemical stability of the NCs.

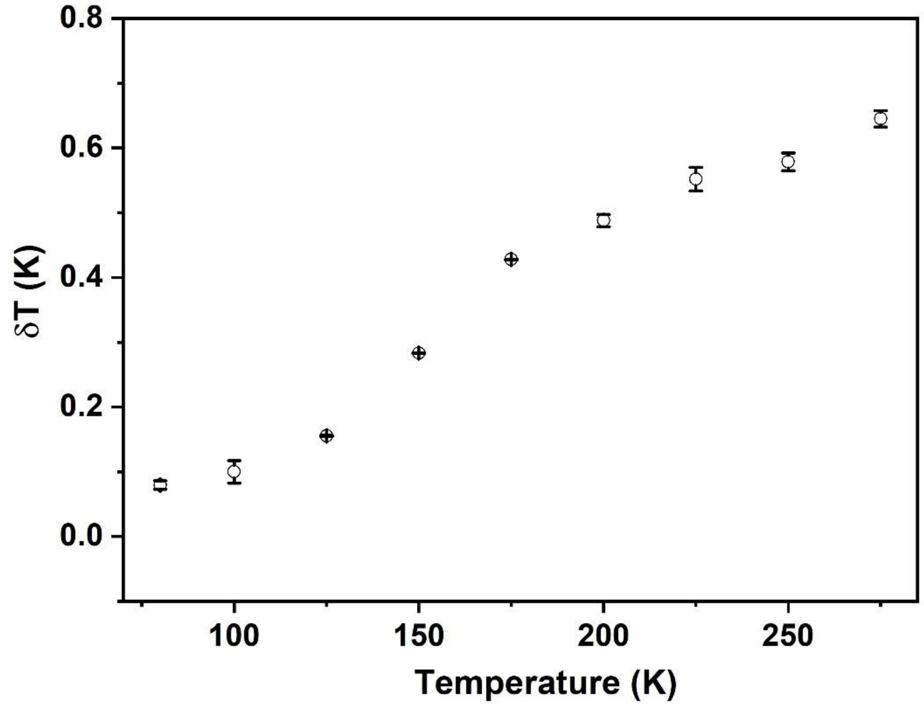

Figure S9. Temperature uncertainty (δT) of $LiLuF_4:Nd^{3+}$ nanothermometer, as calculated by the equation: $\delta T = \frac{1}{S_r}\frac{\delta R}{R}$, where S_r is the relative temperature sensitivity and $\frac{\delta R}{R}$ is the relative error of the response.[1, 10] The error bars result from error propagation in the determination of the temperature uncertainty by the equation.

References

[1] S. Balabhadra, M. L. Debasu, C. D. S. Brites, L. A. O. Nunes, O. L. Malta, J. Rocha, M. Bettinelli, L. D. Carlos, Nanoscale **2015**, 7, 17261.

[2] M. Pedroni, P. Cortelletti, I. X. Cantarelli, N. Pinna, P. Canton, M. Quintanilla, F. Vetrone, A. Speghini, Sensor. Actuat. B-Chem. **2017**, 250, 147.

[3] I. E. Kolesnikov, M. A. Kurochkin, A. A. Kalinichev, D. V. Mamonova, E. Y. Kolesnikov, A. V. Kurochkin, E. Lähderanta, M. D. Mikhailov, J Alloy Compd **2018**, 734, 136.

[4] E. Carrasco, B. del Rosal, F. Sanz-Rodríguez, Á. J. de la Fuente, P. H. Gonzalez, U. Rocha, K. U. Kumar, C. Jacinto, J. G. Solé, D. Jaque, Adv. Funct. Mater. **2015**, 25, 615.

[5] L. Marciniak, K. Prorok, A. Bednarkiewicz, A. Kowalczyk, D. Hreniak, W. Strek, J. Lumin. **2016**, 176, 144.

[6] A. Skripka, V. Karabanovas, G. Jarockyte, R. Marin, V. Tam, M. Cerruti, R. Rotomskis, F. Vetrone, Adv. Funct. Mater. **2018**, 1807105, DOI: 10.1002/adfm.201807105.

[7] M. Quintanilla, Y. Zhang, L. M. Liz-Marzán, Chem. Mater. **2018**, 30, 2819.

[8] O. Savchuk, J. J. Carvajal, L. G. De la Cruz, P. Haro-González, M. Aguiló, F. Díaz, J. Mater. Chem. C **2016**, 4, 7397.

[9] D. Wawrzynczyk, A. Bednarkiewicz, M. Nyk, W. Strek, M. Samoc, Nanoscale **2012**, 4, 6959.

[10] Z. Wang, D. Ananias, A. Carné-Sánchez, C. D. S. Brites, I. Imaz, D. Maspoch, J. Rocha, L. D. Carlos, Adv. Funct. Mater. **2015**, 25, 2824.
